# Resolving heterogeneity in Lymph Node Stromal Cells using high-dimensional analysis of non-optimized flow cytometry data

**DOI:** 10.3389/fbinf.2026.1657030

**Published:** 2026-04-14

**Authors:** Mikala E. Heon, Eduardo Rosa-Molinar

**Affiliations:** 1 The University of Kansas, Lawrence, KS, United States; 2 Bioengineering Program, Lawrence, KS, United States; 3 Department of Pharmacology, Toxicology, Neuroscience, and Bioengineering Program, Lawrence, KS, United States; 4 Departments of Cell Biology & Physiology and Neuroscience, Washington University in Saint Louis School of Medicine, Saint Louis, MO, United States; 5 Brain Tumor Center at Siteman Cancer Center, Saint Louis, MO, United States; 6 Washington University Center for Cellular Imaging, Saint Louis, MO, United States

**Keywords:** bioimage informatics, fibroblastic reticular cells, flow cytometry, heterogeneity, lymph node, machine learning, stromal cells

## Abstract

Lymph Node Stromal Cells (LNSCs) are a diverse population of cells responsible for maintaining the lymph node environment and regulating the immune response. Given these roles, they have the potential to help replicate lymph node functions *invitro*. However, LNSCs are challenging to work with due to their high heterogeneity. Here, we demonstrate the challenges of working with heterogeneous cell populations, where ratios between populations can change over time. We show how similar marker expression profiles between populations, along with non-optimized controls due to experimental limitations, can make flow cytometry analysis difficult. To better assess this heterogeneous population, we demonstrate how to use machine learning algorithms to identify changing populations while overcoming the limitations of any single algorithm. This approach reduces the effects of user bias when placing gates while also increasing confidence in population identification. This analysis method is robust, utilizes existing tools, and provides information that can inform various directions of future studies.

## 1 Introduction

Lymph Node Stromal Cells (LNSCs) play a crucial role in maintaining the structure and function of the lymph node. Each of the four main types plays an important role. Fibroblastic Reticular Cells (FRCs) create and maintain the reticular network and direct immune cell migration; Lymphatic Endothelial Cells (LECs) line sinuses and lymphatic capillaries and direct immune cell migration; Blood Endothelial Cells (BECs) form blood vessels throughout the lymph node and also direct immune cell migration; and Perivascular Cells (PvCs) wrap around blood vessels and help maintain vessel stability and function. Each of these populations, except PvCs, has been found to be much more heterogeneous than previously thought, with each subtype having a specific role in a specific area of the lymph node (Grasso et al., 2021).

Having a diverse population of cells with many different specialties could be extremely beneficial for growing a tissue *invitro*, especially if communication among the different cell types might aid self-organization (Tomei et al., 2009; Onder et al., 2017; Badillo et al., 2020; Krishnamurty and Turley, 2020). When establishing a heterogeneous cell line, it is essential to understand the types of populations present, the number of each type of cell, and how their ratio changes over time. Without this understanding, reproducibility will suffer.

In this work, we aimed to authenticate the cells by protein expression rather than relying solely on genomic or STR profiles. We chose flow cytometry for its ability to show protein expression at the level of individual cells as well as across the population as a whole. This would allow us to closely monitor our heterogeneous population and accurately detect shifts between subpopulations. Flow cytometry is a widely used method for characterizing cells and is reported in studies throughout the biological literature. However, it is deceptively simple and often performed by non-experts. Preparing and running samples in a core laboratory is relatively straightforward. Deciding on controls is often influenced by word-of-mouth or brief summaries in a methods section. Analysis appears simple, especially with the increasing availability of tools to expedite the process. Best practices and a true understanding of how tools impact data analysis are often overlooked.

Due to these factors, data can be messy, especially in highly heterogeneous cell populations, such as LNSCs, where marker expression may be similar across subpopulations. According to Rodda et al. and Grasso et al., much of the heterogeneity of LNSCs has been identified using more markers than can be assessed in a typical flow cytometry assay (Rodda et al., 2018; Grasso et al., 2021). In this work, we used only five markers and aimed to glean as much information about heterogeneity as possible to inform the direction of further analysis. Traditional gating methods examine the data in one or two dimensions at a time and rely on clear separation between populations for accurate gate placement. When populations have similar marker expression, such as in our heterogeneous populations, gate placement is difficult.

With the rise of machine learning, new algorithms have been developed to analyze flow cytometry data across more than 2 dimensions simultaneously. High-dimensional analysis can increase separation between populations and make gating easier. Machine learning algorithms can identify populations quickly and may reveal smaller populations that might otherwise be missed. However, there is a steep learning curve to master these tools. This learning curve creates a barrier to use and will likely result in the algorithms being used without full understanding or optimization. Results will likely be trusted blindly without understanding what is and is not biologically significant.

Here, we demonstrate how high-dimensional analysis tools can be used by non-experts to identify populations in datasets with substantial signal overlap. To simplify the process, we examine patterns and compare results from a few algorithms to guide gate placement. Because each algorithm has its own strengths and weaknesses, comparing their results helps distinguish what is real from what might be an artifact. This approach is easy to follow, less prone to user bias than traditional gating, and will help improve rigor and reproducibility for anyone working with a heterogeneous population of cells.

## 2 Results

### 2.1 Limitations put on flow cytometry controls when working with a small population

The Lymph Node Stromal Cell line analyzed in this work was isolated from postmortem murine inguinal lymph nodes of three mice. Due to the small size of these organs, the number of cells isolated and immortalized was relatively small, far from the millions needed for flow cytometry. This low initial cell count meant the cells could not be analyzed immediately after immortalization. Instead, their population was expanded over several passages, and a compromise had to be made between analyzing them at a low passage number and having a sufficient number of cells for robust controls. Additionally, due to a long and unpredictable travel time between our lab and the flow cytometer, we wanted to limit the number of samples to prepare and thus the amount of time the cells were under stress.

We would like to emphasize that although best practices for flow cytometry controls have been outlined (Maecker and Trotter, 2006; Hulspas et al., 2009; Cossarizza et al., 2017), it is still common to find papers in which these controls were overlooked, not reported, or misunderstood. Thus, although the compromises described here were intentional, it is not unreasonable to assume that others have performed flow cytometry experiments before understanding the importance of controls for interpreting their results.

Blocking controls, in which a cell sample is incubated with serum to block antibody-binding sites and assess non-specific antibody binding, require additional cell samples. Due to the limited number of cells available at low passages, we were unable to include these controls. Another way to assess non-specific binding and cross-reactivity would have been to use a nearly identical cell population that is negative for the markers. This type of control was also unavailable to us. Thus, when interpreting our results, we need to consider the possibility of cross-reactivity and non-specific antibody binding, and in future studies invest in antibody validation.

To assess background fluorescence from autofluorescence or spectral overlap among fluorophores, best practice is to include Fluorescence-Minus-One (FMO) controls. However, due to the limited number of cells, we could not include FMO cell controls. Instead, we included a cell sample with no antibody labeling to account for cell autofluorescence and single-labeled compensation beads to address spectral overlap, with the understanding that using FMO controls in the future could improve data clarity. We repeated these controls on both days we ran samples to help detect and compensate for batch effects.

### 2.2 Engineer’s approach to normalization

When samples are run on different days, it is possible to observe variation in the data due to external factors, known as batch effects. By preparing compensation beads alongside our samples each day, we identified a significant batch effect, particularly in the signal from the ICAM-1 antibody. To correct these batch effects, we first evaluated three commonly used normalization algorithms: CytoNorm, CyCombine, and MNN.

CytoNorm works by mapping the data to a FlowSOM spline and corrects the data using common quantiles (Van Gassen et al., 2020) and thus requires the control samples to cover the entire range and to have a distribution roughly similar to that of the experimental samples. Our compensation bead controls consist of two sharp peaks representing positive and negative signals and were therefore insufficient for use with the CytoNorm algorithm.

**FIGURE 1:**
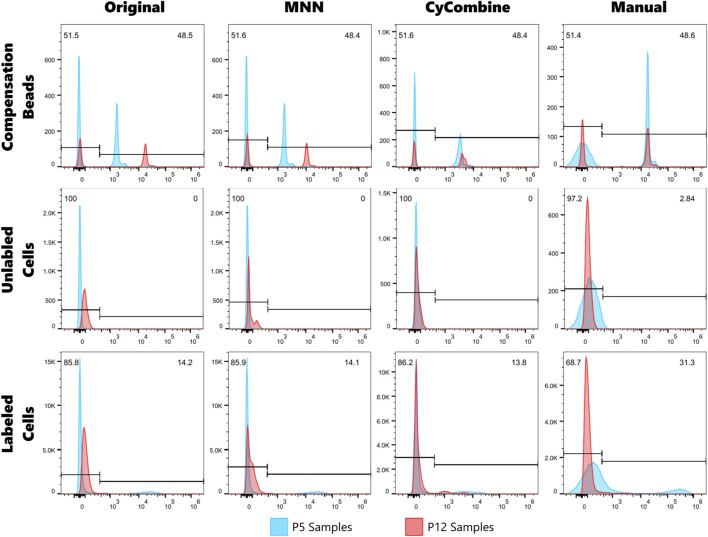
Histograms of ICAM-1 signal comparing the original data to data after normalization for Compensation Beads, Unlabeled Cells, and Labeled Cells. Three normalization methods are shown: MNN, CyCombine, and our manual method. Gates are identical on all plots. Numbers in the top corners represent the percentage of P5 events which fall on either side of the gate.

MNN groups cells by Euclidean distance, then looks for the nearest group of cells in other batches. This process relies on the assumption that differences between cell populations are greater than differences between batches (Haghverdi et al., 2018). In our case, the difference in ICAM-1 signal is so large that MNN could not fully correct it (Figure 1).

CyCombine is more robust than CytoNorm with respect to sample requirements and overcomes the assumptions of MNN. It can rank the expression of each marker for each cell and then cluster cells based on similar rankings (Pedersen et al., 2022). Of these three algorithms, CyCombine performed best but still struggled to align the positive compensation bead peaks without distorting the positive distributions.

**FIGURE 2:**
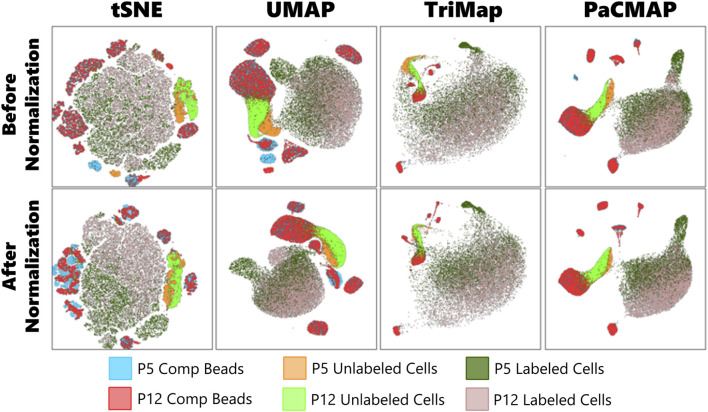
Comparison of dimension reduction plots before and after normalization using four dimension reduction algorithms: tSNE, UMAP, TriMap, and PaCMAP.

Unfortunately, our ICAM-1 channel exhibited a larger batch effect than these algorithms could accurately account for, and after a preliminary run through dimensionality reduction, it was clear this discrepancy would affect our results (Figure 2). To address this issue, we derived an equation to manually normalize our data.

**FIGURE 3:**
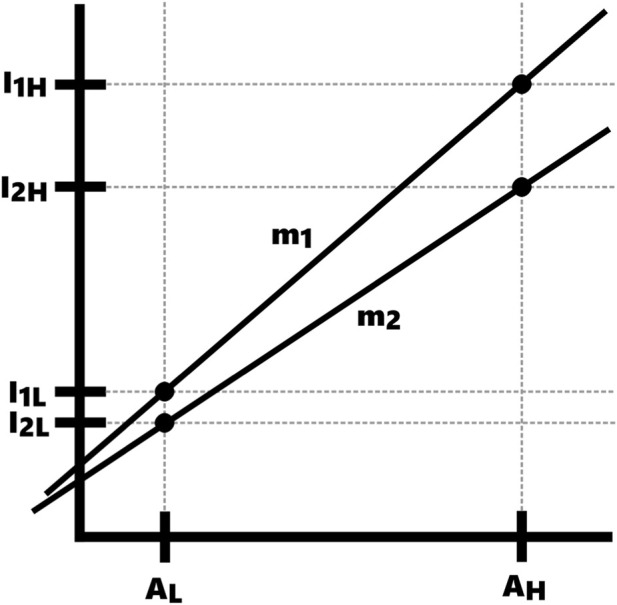
Illustration of the concepts used for normalization. *A* denotes the number of bound antibodies, *I* denotes the measured intensity. *L* represents the lower peak while *H* represents the higher peak. *m* is the slope between the points.

We know that each intensity measurement corresponds to a certain amount of antibody and that the relationship between the two is roughly linear at low concentrations (Figure 3). To use this assumption, we previously titrated the antibodies to determine the lowest concentration that would produce a sufficient signal.

Therefore, we can write out linear equations for each day:

Day 1: $I_{1L}=m_{1}A_{1L}+b_{1}$ and $I_{1H}=m_{1}A_{1H}+b_{1}$

Day 2: $I_{2L}=m_{2}A_{2L}+b_{2}$ and $I_{2H}=m_{2}A_{2H}+b_{2}$

Where I is the measured intensity, m is the slope of the line for that day, A is the number of antibodies, and b is the y-intercept. L denotes the low peak and H denotes the high peak. Using these equations, we can solve the slopes:

$$m_{1}=\frac{I_{1H}-I_{1L}}{A_{1H}-A_{1L}}$$

$$m_{2}=\frac{I_{2H}-I_{2L}}{A_{2H}-A_{2L}}$$

Because we are looking at compensation beads, which should have no variability between days, we can assume that the number of antibodies is the same on both days. $A_{1}=A_{2}$ . Finishing solving for the equations, we get

$$I_{1}=m_{1}\left(A-A_{L}\right)+I_{1L}$$

$$I_{2}=m_{2}\left(A-A_{L}\right)+I_{2L}$$

In theory, the negative beads in the compensation controls should have no antibodies bound to them, therefore $A_{L}\to 0$ . Thus, we get:

$$I_{1}=m_{1}A+I_{1LC}$$

$$I_{2}=m_{2}A+I_{2LC}$$

Where $I_{1LC}$ and $I_{2LC}$ represent the median or mean of the lower peak of the controls on Day 1 and Day 2 respectively. From here, we can solve for A:

$$A=\frac{I_{2}-I_{2LC}}{m_{2}}$$

∴

$$I_{1}=\frac{m_{1}}{m_{2}}\left(I_{2}-I_{2LC}\right)+I_{1LC}$$

Writing everything out to normalize Day 2 to Day 1, we get:

$$I_{1}=\left(\frac{I_{1HC}-I_{1LC}}{I_{2HC}-I_{2LC}}\right)\left(I_{2}-I_{2LC}\right)+I_{1LC}$$

Where $I_{1HC}$ is the mean or median of the higher control peak of Day 1, $I_{1LC}$ is the mean or median of the lower control peak of Day 1, $I_{2HC}$ is the mean or median of the higher control peak of Day 2, $I_{2LC}$ is the mean or median of the lower control peak of Day 2, and $I_{2}$ is the measured parameter that needs to be normalized. We compared using both mean and median values and found not much difference between the two. We chose to use the normalized values based on the median for the rest of this experiment (Supplementary Figure S1).

The biggest drawback of normalizing the data with this method is that it acts as a multiplier, forcing the positive peaks to align and thereby amplifying the distribution, which negatively impacts future statistical analysis. Because our data spans orders of magnitude, the most significant effect will be seen at very low values. Our negative controls and samples will be affected much more than positive populations (Figure 1). We normalized the day with a lower signal to the day with a higher signal, which increased our distribution. If we had gone in the opposite direction, our distribution would have decreased.

### 2.3 Gating populations using traditional gating methods

Traditionally, cell populations in flow cytometry data are identified by gating on one- or two-dimensional plots, with each axis representing a marker of interest. When more than two markers are used to identify populations from a sample, we must prioritize marker combinations and use successive rounds of gating to further distinguish populations. We will refer to these successive rounds as gating “layers”. In our example, we first gated on CD45 expression to remove CD45^+^ cells. Next, we separated the cells by CD31 and PDPN expression, as is typical for identifying lymph node stromal cells. Lastly, we attempted to identify subpopulations based on ICAM-1 and VCAM-1 expression. Thus, to analyze our cells using five markers, we needed at least three gating “layers”.

**FIGURE 4:**
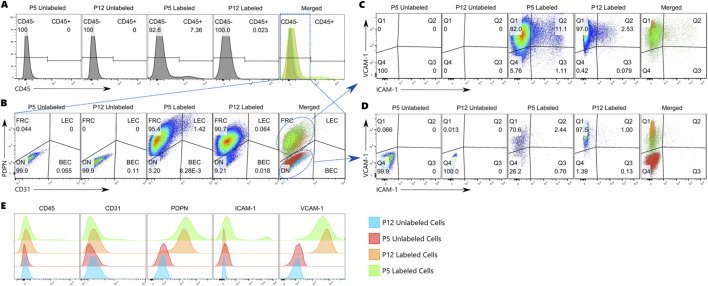
**(A)** CD45 histograms showing positive and negative gating; **(B)** CD31 and PDPN expression of the CD45- population with gates separating the four main LNSC populations; **(C)** ICAM-1 and VCAM-1 expression of the FRC population; **(D)** ICAM-1 and VCAM-1 expression of the PvC (DN) population; **(E)** Histograms of the overall expression for each sample. Numbers denote the percentage of events that fall within each gate.

When gating on the CD45 histograms, we set the gate as low as possible while ensuring that at least 99.9% of both unlabeled cell samples remained within the negative gate (Figure 4A; Supplementary Figure S3). This resulted in 7.36% of the Labeled P5 sample falling into the CD45^+^ gate and only 0.023% of the Labeled P12 sample. Although there are clearly two peaks, the curve between them is relatively flat, likely due to overlapping signals between the populations. This overlap makes exact gate placement difficult and subject to user interpretation. To demonstrate the effect of this issue, we repeated CD45 gating three times using different criteria to simulate user variation: 1) following the above strategy, 2) setting the gate as low as possible with 100% of the unlabeled cells within the negative gate, and 3) setting the gate so that 99.5% of the unlabeled cells fell within the negative gate. The resulting CD45^+^ populations ranged from 4.55% of all P5 cells to 4.08% of all P12 cells, indicating that seemingly small changes in gate placement can have a significant effect on final population size (Supplementary Figure S2).

The second “layer” of gating analyzes cells that fell within the CD45^−^ gate of the first “layer”. By examining two markers simultaneously, we can view the issue described above from a different perspective (Figure 4B; Supplementary Figure S4). If we were to look only at the PDPN histogram, we would see significant overlap between positive and negative populations and have difficulty accurately placing the gate. By including a second parameter, the CD31 marker, the populations can be more clearly distinguished. We drew gates around the negative population using diagonal lines to retain 99.9% of the unlabeled cells. However, gating the upper right quadrant remained challenging without known single- or double-positive cell populations. Gating the double-positive quadrant thus relied on prior knowledge or assumptions about our population. Given the high degree of signal overlap and the presumed heterogeneity of our samples, we understood that accurate gate placement would be nearly impossible. Initial gate placement indicated that 95.4% of all CD45^−^ P5 cells and 90.7% of all CD45^−^ P12 cells were FRCs. As before, we repeated gate placement using three strategies: 1) mimic the original gating, 2) minimize the FRC population, 3) maximize the FRC population. The resulting population ranges averaged 1.38% for LECs, 4.35% for PvCs, and 3.4% for FRCs (Supplementary Figure S2). The resulting cell counts in each population had an average covariance of 29% (Supplementary Tables S1, S2).

To investigate heterogeneity within each LNSC population, we examined ICAM-1 and VCAM-1 expression in our double-negative (DN)/perivascular cell (PvC) population and FRC population (Figures 4C,D; Supplementary Figure S5). We encountered the same challenges described above when placing gates, especially in the double-positive quadrant. Although we expected gating variations to be similar to those on the CD31/PDPN plots, we also recognized that population sizes determined at this “layer” would be affected by variations in previous “layers”. In other words, variation compounds with each successive “layer” of gating, making the final population size highly subjective to user bias and assumptions. If our populations were clearly distinguishable, there would be little impact on our results. However, given the high heterogeneity of our population and the overlap of signal expression along any one dimension (Figure 4E), we needed to consider analyzing our samples by examining all five markers at once.

### 2.4 Dimension reduction and sample combinations

To compare our data across all five dimensions at once, we needed to perform dimension reduction. Dimension reduction is the process of taking data from a high-dimensional space, such as when several parameters are considered together, and projecting it onto a 2D plot. Many strategies are used to create these projections, and each prioritizes mapping distances between nearby points (local structure) versus overall distances between groups (global structure) differently (Huang et al., 2022). For example, tSNE is known for providing an in-depth view of the local structure of the data (Van der Maaten and Hinton, 2008), while UMAP strikes a better balance between local and global structure (McInnes et al., 2018). TriMap is known for showing relationships between populations on the global scale (Amid and Warmuth, 2019), and PaCMAP prioritizes both local and global structure (Wang et al., 2021). Choosing which algorithm to use can be complicated and often requires prior knowledge of the cell population. While it is possible to blindly pick a single algorithm and adjust parameters until expected populations are clearly distinguished, this process takes a lot of time (Supplementary Table S3), careful attention to user bias, and a level of expertise beyond that of non-expert users. To overcome these obstacles, we chose to run four commonly used dimension reduction algorithms at their default parameters and compare common patterns among them.

**FIGURE 5:**
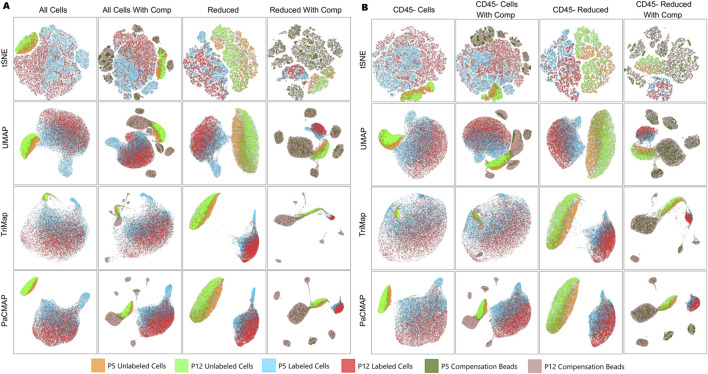
Comparison of dimension reduction algorithms: **(A)** with all five parameters considered and **(B)** without including the CD45 parameter. Columns correspond to datasets with varying combinations of sample types.

Performing dimension reduction first requires combining all sample data sets into a single file with identical parameters. The scales for each parameter need to be transformed to maximize separation between positive and negative populations while keeping data points within the boundaries of analysis, meaning not stacking at the graph edges. The type of sample, number of cells, and populations included can also affect the outcome of dimension reduction. For our population, we compared three variables when deciding which data to include in the analysis: 1) Whether to include CD45^+^ cells, because this population is so small that this channel will be mostly noise; 2) Whether to include compensation beads that might act as a benchmark for positive and negative signals; 3) Whether to limit the sample size so that each sample type has the same number of events (Figure 5). As a result, we analyzed eight data sets described in Table 1, using the four dimension reduction algorithms on each.

**TABLE 1:** Sample combinations used in comparison of dimension reduction algorithms. None: none of the sample used. All: all events in the sample used. Limited: number of events used from each sample is equal to the smallest sample size. Gated CD45^-^: only events falling within the CD45^−^ gate are used.

Dataset name	Compensation beads	Unlabeled cells	Labeled cells
All cells	None	All	All
All cells with comp	All	All	All
Reduced	None	Limited	Limited
Reduced with comp	Limited	Limited	Limited
CD45^−^ cells	None	Gated CD45^−^	Gated CD45^−^
CD45^−^ cells with comp	Gated CD45^−^	Gated CD45^−^	Gated CD45^−^
CD45^−^ reduced	None	Gated CD45^−^, limited	Gated CD45^−^, limited
CD45^−^ reduced with comp	Gated CD45^−^, limited	Gated CD45^−^, limited	Gated CD45^−^, limited

We found that removing CD45^+^ cells from our analysis and excluding that parameter increased the resolution of the remaining clusters (Figure 5B). We decided to use samples with all cells and parameters to assess overall composition, and samples with only CD45^−^ cells to help identify heterogeneity in the LNSCs. Including compensation bead samples increased the resolution of the cell samples and provided benchmarks for assessing clustering and for drawing our own gates. Reducing sample size so that each sample type contained the same number of events decreased population separation and made it difficult to visualize the labeled cell samples. We also had concerns that limiting the number of cells would make it difficult to see rarer or smaller populations. For these reasons, we decided to use two sample combinations using all events and compensation beads: one with all CD45^+^ cells and the parameter, and one without.

### 2.5 Machine learning identification of populations

The dimension reduction plots allow us to gate our samples using all five parameters at once; however, overlap between populations made gate placement unclear. Thus, we examined machine learning clustering algorithms to identify mathematically significant populations. Three algorithms were considered: FlowSOM, Phenograph, and X-Shift. FlowSOM uses a self-organizing map to match cells to clusters of the most similar phenotype and is the most conservative of these three algorithms (Van Gassen et al., 2015). It relies on a user-inputted estimate of the number of populations. Phenograph and X-Shift both rely on a nearest-neighbor graph to find areas of high density and require no prior knowledge of the number of populations. Phenograph uses a community detection algorithm to create clusters of cells in dense areas of the graph (Levine et al., 2015). X-Shift assigns cells to clusters by following a density gradient to the nearest density peak and is best known for its ability to find rare populations (Samusik et al., 2016).

When using any of these algorithms, one should be familiar with how they work, optimize parameters for each data set, and understand their limitations. This step often requires the *most* care to avoid bias and to question results. It is easy for non-expert users to overlook these aspects and draw false conclusions, especially when results appear exciting or confirm hypotheses. When parameters are not optimized or data sets have significant overlap between populations, such as in our samples, these algorithms are likely to identify clusters within normal biological variation. To understand how these algorithms handled our samples, we used their default parameters and compared the results to our known sample types and controls.

**FIGURE 6:**
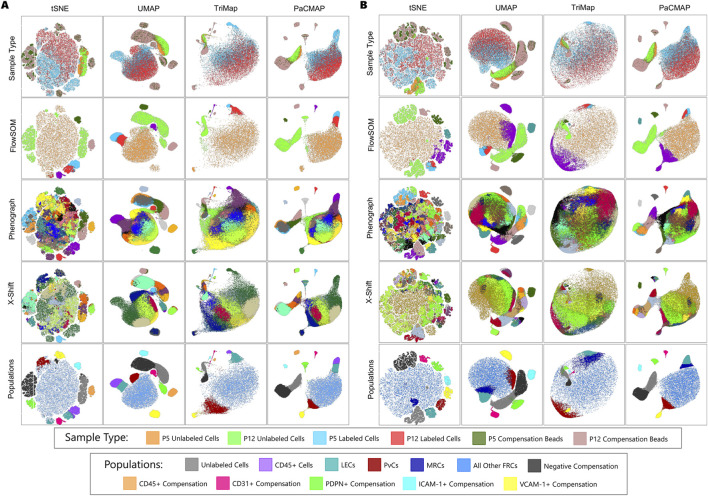
Comparison of clustering methods for **(A)** All Cells with Compensation Beads and **(B)** CD45^−^ Cells with Compensation Beads. Colors in the top row denote “Sample Type”. Colors in the FlowSOM, Phenograph, and X-Shift rows denote clusters identified by the respective algorithm. Colors in the bottom row denote “Populations”.

FlowSOM was the only one of the three algorithms to cluster all known negative samples into a single population. It was also the most conservative, reporting only seven distinct populations. In the dataset containing all events, it correctly identified the ICAM-1^+^, CD45^+^, and CD31^+^ compensation beads as distinct populations; however, it grouped the PDPN^+^ compensation bead population into the negative group and the VCAM-1^+^ population with the bulk of the labeled cells (Figure 6A). When CD45^+^ cells were removed and that parameter ignored, FlowSOM successfully clustered all of the compensation beads into their correct populations, except for the VCAM-1^+^ beads, which remained grouped with some of the labeled cells (Figure 6B).

Both Phenograph and X-Shift divided the unlabeled cells and negative compensation beads into several populations that were not biologically significant. Phenograph was the only algorithm to successfully cluster all positive compensation beads into their own distinct populations. X-Shift had difficulty distinguishing the VCAM-1^+^ compensation beads from the bulk of the cells while also dividing all other positive compensation beads into multiple populations (Figure 6).

After observing how these three algorithms clustered our known populations and controls, it was clear we could not trust their results outright. Instead, we needed to conduct our own analysis to help narrow down what was and was not biologically significant.

### 2.6 Gating our own populations

Although the machine learning clustering algorithms failed to correctly group known populations and controls, the patterns among their results provided valuable insight into where we could confidently draw our own gates. To guide these decisions, we used Euclid (Hartigan, 1985), a FlowJo plugin, to assess which clusters should be split into two or more populations and which clusters are sufficiently similar to be combined into a single population. We also visually compared the placement of clusters across all four types of dimension reduction plots, checking for algorithmic similarities, population overlap, and alignment with our known populations.

The first gates were drawn around the most easily distinguishable populations: the positive compensation beads. For each population, at least one dimension reduction plot contained it as a single, isolated island. We selected the plot with the clearest delineation and the least scatter to draw the gates (Figure 6: “Populations” Orange, Pink, Green, Bright Teal, and Yellow). The populations were identified by looking for a positive signal in the parameter histograms.

**FIGURE 7:**
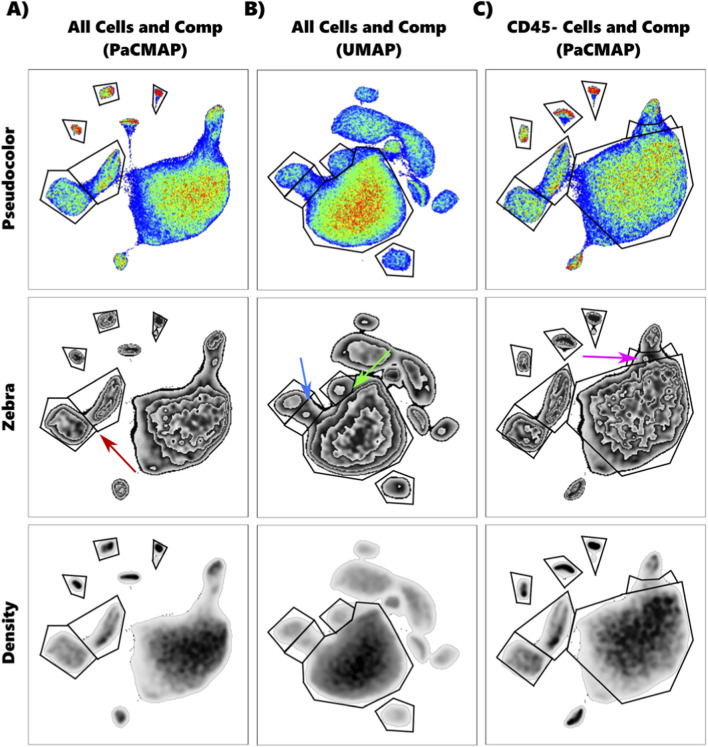
Example plots to demonstrate manual gating strategy using three views: Pseudocolor, Zebra, and Density plots. **(A)** PaCMAP plot of the “All Cells and Compensation Beads” dataset showing an isolated island of the negative populations with a low-density area between lobes where the gate was drawn (Red Arrow). **(B)** UMAP plot showing where the gate was drawn between “two hills” (Blue Arrow). Green Arrow shows where a similar “hill” was observed but with greater connection to the main island. **(C)** PacMAP plot of the “CD45^-^ Cells and Compensation Beads” dataset showing the unclear placement of the MRC gate (Pink Arrow).

Next, we examined the negative populations: the unlabeled cells and negative compensation beads. These populations were split into two lobes of a distinct island on most of the dimension reduction plots. Gate placement was determined by selecting the dimension reduction plot with the clearest distinction and drawing a line between the lightest zone of the Zebra plot (Figure 7A).

The labeled cell populations were the most difficult to discern; we relied on the clustering algorithms to guide decision-making. In the UMAP, TriMap, and PaCMAP plots, there was a lobe that all clustering algorithms aligned with a distinct island in the tSNE plot. All algorithms indicated that cells in this lobe were different from the bulk of the cells. While X-Shift clustered this lobe as a single population, both FlowSOM and Phenograph split it into two separate populations, with a similarly placed line between them. In the UMAP Zebra plot, two distinct “hills” were visible (Figure 7B), and TriMAP showed a narrowing between them. These observations allowed us to confidently gate these cells as two distinct populations (Figure 6: “Populations” Purple and Dark Teal).

The UMAP Zebra plot showed a similar “hill” corresponding to clusters identified by Phenograph and X-Shift (Figure 7B). Unlike the previous populations, this one had no clear boundary to draw a line between it and the rest of the labeled cells, so we followed the line drawn by Phenograph and X-Shift as best we could (Figure 6: “Populations” Red).

This same strategy was applied to the data file that lacked a CD45 parameter. In this file, a similar lobe was observed off the bulk of the cells in the UMAP, TriMap, and PaCMAP plots. Again, all three clustering algorithms showed that these cells corresponded to a distinct island on the tSNE plot. However, unlike before, the clustering algorithms disagreed on how to divide the lobe, though FlowSOM and Phenograph both indicated two clusters. We decided to divide the lobe into two separate populations, with the understanding that this last gate (Figure 6B: “Populations” Dark Blue) is likely inaccurately placed and blends into the populations nearby (Figure 7C).

**FIGURE 8:**
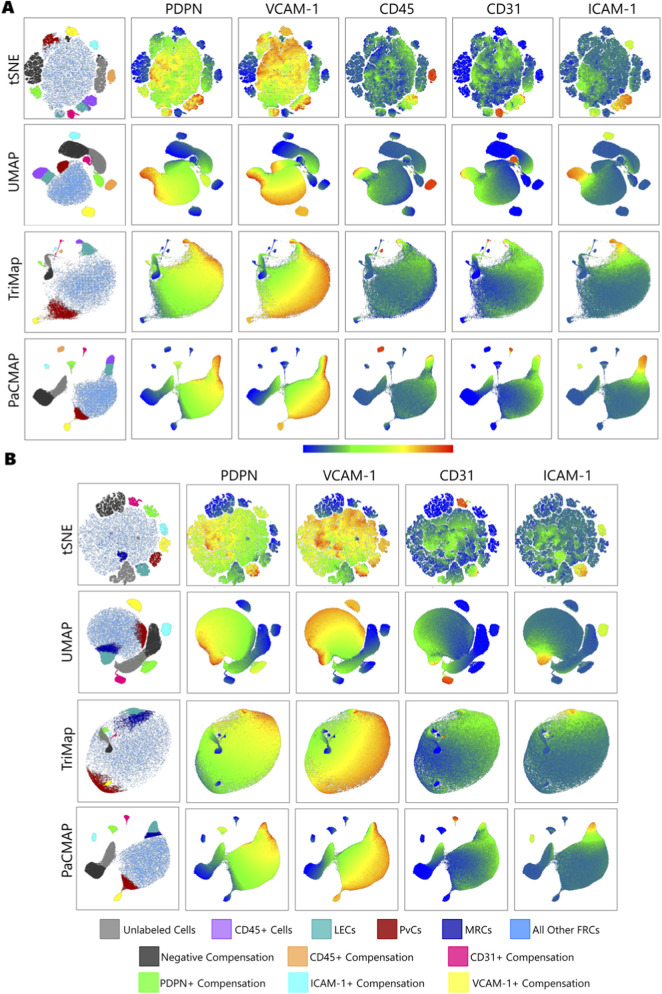
Heatmaps of each dimension reduction plot showing CD45, CD31, PDPN, ICAM-1, and VCAM-1 signal. **(A)** All Cells with Compensation Beads, **(B)** CD45^−^ Cells with Compensation Beads. Legend corresponds to the first columns.

The remaining populations identified by the clustering algorithms all fall within the bulk of the labeled cells (Figure 6: “Populations” Blue). FlowSOM clustered all these cells together, while Phenograph and X-Shift recognized them as many distinct populations. A close examination of these clusters made clear that there is extensive overlap among them, with no clear place to draw a gate with any confidence. In the heatmaps (Figure 8), we observed a wide range of marker expression throughout this group, indicating that there are likely multiple subpopulations within. However, we also recognized that we could not clearly resolve these populations with the limited markers used, and it would be unwise to try to gate them. We thus left it as a single population.

### 2.7 Identifying and validating our populations

We identified 11 populations: 1 unlabeled cell population, 1 negative compensation bead population, 5 positive compensation bead populations, and 4 labeled cell populations. We identified each population by comparing its expression levels to those of the unlabeled cells. A significant expression level was confirmed by a high Chi-Squared T(x) score using the population comparison tool built into FlowJo™ v.10.10.0 (Roederer et al., 2001a; Roederer et al., 2001b). We also compared Chi-Squared T(x) scores among our populations to ensure that each was statistically distinct from all the others.

When running our analysis, we chose to exclude the high ICAM-1 control T(x) because the T(x) value for the negative compensation beads was significantly greater than the biological variability in the unlabeled cell control (Table 2). Because we understood that this increased spread is due to the mathematical transformation of the data and that the effects are less significant at the higher values where we see positive signal, we felt comfortable excluding the negative compensation beads from our analysis.

**TABLE 2:** Chi-Squared T(x) scores for individual channels on the sample with all cells.

T(x) score of controls						
P12 vs. P5	CD45	CD31	PDPN	ICAM-1	ICAM-1*	VCAM-1
CD45^+^ comp	11.3249	27.6054	0.0000	236.1523	236.1523	2.6107
CD31^+^ comp	6.7326	100.1091	0.0000	123.2704	123.2704	0.0000
PDPN^+^ comp	8.1574	25.0842	21.0292	343.4918	343.4918	0.0000
ICAM^+^ comp	0.0000	2.4131	0.0000	0.0000	0.0000	0.0000
VCAM^+^ comp	0.0229	36.1395	0.0000	372.5375	372.5375	0.0000
Negative comp	38.4676	115.2505	0.7437	1,686.9919		16.1258
Unlabeled cells	41.3859	146.3102	81.1434	713.0674	713.0674	128.4021
*MAX*	41.3859	146.3102	81.1434	1,686.9919	713.0674	128.4021

Original T(x) score						
Unlabeled cells vs.	CD45	CD31	PDPN	ICAM-1	ICAM-1*	VCAM-1
Population 1	1907.0868	1728.4850	1910.5112	1911.0552	NA	1824.3408
Population 2	686.1364	601.5001	1,392.0356	1,390.4099	NA	1,302.1653
Population 3	188.7852	54.7781	39.1662	223.1648	NA	1,549.5736
Population 4	549.2644	19.1622	4,344.3086	238.9766	NA	4,539.8711

Population 4 vs.	CD45	CD31	PDPN	ICAM-1	ICAM-1*	VCAM-1
Population 1	1892.2092	1702.9343	1,387.932	1911.0066	NA	169.9426
Population 2	382.5653	546.8763	714.8235	1,383.2584	NA	175.5464
Population 3	35.5171	79.6510	1,498.899	298.5937	NA	152.0607

Population 3 vs.	CD45	CD31	PDPN	ICAM-1	ICAM-1*	VCAM-1
Population 1	1,553.572	1,525.4178	1,551.873	1,556.229	NA	287.5797
Population 2	438.2617	780.4340	1,386.522	1,389.9067	NA	323.1766

Population 2 vs.	CD45	CD31	PDPN	ICAM-1	ICAM-1*	VCAM-1
Population 1	1,143.6311	510.2698	180.2152	608.6154	NA	4.4485

T(x) score minus control						
Unlabeled cells vs.	CD45	CD31	PDPN	ICAM-1	ICAM-1*	VCAM-1
Population 1	1865.7009	1,582.1748	1829.3678	224.0633	1,197.9878	1,695.9387
Population 2	644.7505	455.1899	1,310.8922	0.0000	677.3425	1,173.7632
Population 3	147.3993	0.0000	0.0000	0.0000	0.0000	1,421.1715
Population 4	507.8785	0.0000	4,263.1652	0.0000	0.0000	4,411.4690

Population 4 vs.	CD45	CD31	PDPN	ICAM-1	ICAM-1*	VCAM-1
Population 1	1850.8233	1,556.6241	1,306.7887	224.0147	1,197.9392	41.5405
Population 2	341.1794	400.5661	633.6801	0.0000	670.1910	47.1443
Population 3	0.0000	0.0000	1,417.7560	0.0000	0.0000	23.6586

Population 3 vs.	CD45	CD31	PDPN	ICAM-1	ICAM-1*	VCAM-1
Population 1	1,512.1865	1,379.1076	1,470.7296	0.0000	843.1616	159.1776
Population 2	396.8758	634.1238	1,305.3783	0.0000	676.8393	194.7745

Population 2 vs.	CD45	CD31	PDPN	ICAM-1	ICAM-1*	VCAM-1
Population 1	1,102.2452	363.9596	99.0718	0.0000	0.0000	0.0000

Columns show Chi-Squared T(x) score when compared on individual channels: CD45, CD31, PDPN, ICAM-1, and VCAM-1. The ICAM-1* column denotes values where the ICAM-1 Negative Compensation Bead control T(x) value was excluded.

**FIGURE 9:**
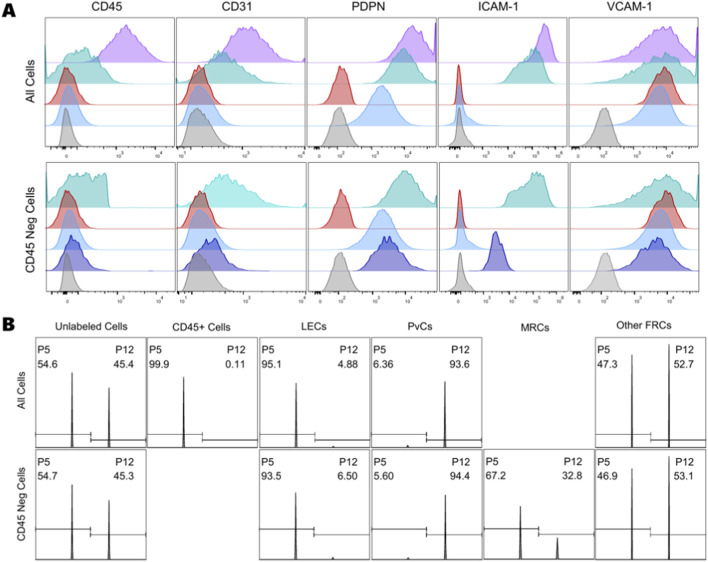
**(A)** Normalized-to-Mode Histograms showing expression of each marker for our identified cell populations. **(B)** Breakdown of how many cells of each population are from either passage number. Purple: CD45^+^ Cells; Teal: LECs; Red: PvCs; Light Blue: FRCs; Dark Blue: MRCs; Gray: Unlabeled Cells.

We found that Population 1 expressed all five markers, indicating that these cells are hematopoietic, likely macrophages or dendritic cells, given their ability to adhere to a culture surface (Table 2). Population 2 was likely Lymphatic Endothelial Cells (LECs), as indicated by expression of CD31^+^, PDPN^+^, ICAM-1^+^, and VCAM-1^+^. Population 3 was positive only for VCAM-1, making them Perivascular Cells (PvCs). Across all cells, Population 4 was likely Fibroblastic Reticular Cells, based on CD45^−^CD31^−^PDPN^+^VCAM-1^+^ expression, although ICAM-1 expression shows an extended tail along the histogram (Figure 9A).

This tail makes sense when examining the data file that excludes CD45^+^ cells, particularly when the extra population is resolved (Table 3). Population 4.1 is PDPN^+^ICAM-1^−^VCAM-1^+^, while Population 4.2 is PDPN^+^ICAM-1^+^VCAM-1^+^. We believe Population 4.2 could be Marginal Reticular Cells (MRCs), a subtype of FRCs found in the subcapsular sinus around the outer areas of the lymph node. However, it is also the only population not statistically significantly different from the two populations it is sandwiched between (Tables 4, 5). This could be influenced by two factors: difficulty gating due to the lack of distinct separation between the populations and a smaller cell number lowering significance. If not for sample size, the Max T(x) for this population could have been 148, which would have indicated a significant difference. So, we believe it is likely that we have MRCs in our sample, but we have not identified them with certainty. Although this is certainly frustrating, it points to the value of adding an additional MRC marker in the future to resolve them clearly.

**TABLE 3:** Chi-squared T(x) scores for individual channels on the sample with CD45^−^ cells.

T(x) score of controls						
P12 vs. P5	CD45	CD31	PDPN	ICAM-1	ICAM-1*	VCAM-1
CD31^+^ comp	6.4353	100.3359	0.0000	123.0754	123.0754	0.0000
PDPN^+^ comp	8.0040	24.8367	20.5766	344.6432	344.6432	0.0000
ICAM^+^ comp	0.0000	2.4131	0.0000	0.0000	0.0000	0.0000
VCAM^+^ comp	0.1650	34.4767	0.0000	374.7589	374.7589	0.0000
Negative comp	23.7439	72.8701	5.4623	1,272.166		7.3756
Unlabeled cells	40.8238	151.5139	85.9399	686.836	686.836	134.3105
*MAX*	40.8238	151.5139	85.9399	1,272.166	686.836	134.3105

Original T(x) score						
Unlabeled cells vs.	CD45	CD31	PDPN	ICAM-1	ICAM-1*	VCAM-1
Population 2	825.0357	757.8271	1,651.3976	1,645.4453	NA	1,542.6506
Population 3	174.0736	48.9193	17.2313	214.5370	NA	1,400.9460
Population 4.1	527.1249	15.0814	4,271.0918	179.5648	NA	4,484.1006
Population 4.2	199.0309	113.9789	903.3472	900.0569	NA	904.6079

Population 4.2 vs.	CD45	CD31	PDPN	ICAM-1	ICAM-1*	VCAM-1
Population 2	169.6603	207.0245	271.5743	848.3762	NA	85.5426
Population 3	78.6708	212.9086	908.3961	937.8569	NA	170.5037
Population 4.1	29.1968	84.5512	75.0648	893.0591	NA	11.7680

Population 4.1 vs.	CD45	CD31	PDPN	ICAM-1	ICAM-1*	VCAM-1
Population 2	483.239	709.8576	854.6854	1,645.4025	NA	193.2527
Population 3	30.7679	65.0197	1,340.9312	272.1054	NA	169.6285

Population 3 vs.	CD45	CD31	PDPN	ICAM-1	ICAM-1*	VCAM-1
Population 2	449.9532	809.0991	1,396.7487	1,391.7103	NA	315.5424

T(x) score minus control						
Unlabeled cells vs.	CD45	CD31	PDPN	ICAM-1	ICAM-1*	VCAM-1
Population 2	784.2119	606.3132	1,565.4577	373.2793	958.6093	1,408.3401
Population 3	133.2498	0.0000	0.0000	0.0000	0.0000	1,266.6355
Population 4.1	486.3011	0.0000	4,185.1519	0.0000	0.0000	4,349.7901
Population 4.2	158.2071	0.0000	817.4073	0.0000	213.2209	770.2974

Population 4.2 vs.	CD45	CD31	PDPN	ICAM-1	ICAM-1*	VCAM-1
Population 2	128.8365	55.5106	185.6344	0.0000	161.5402	0.0000
Population 3	37.8470	61.3947	822.4562	0.0000	251.0209	36.1932
Population 4.1	0.0000	0.0000	0.0000	0.0000	206.2231	0.0000

Population 4.1 vs.	CD45	CD31	PDPN	ICAM-1	ICAM-1*	VCAM-1
Population 2	442.4152	558.3437	768.7455	373.2365	958.5665	58.9422
Population 3	0.0000	0.0000	1,254.9913	0.0000	0.0000	35.3180

Population 3 vs.	CD45	CD31	PDPN	ICAM-1	ICAM-1*	VCAM-1
Population 2	409.1294	657.5852	1,310.8088	119.5443	704.8743	181.2319

Columns show Chi-Squared T(x) score when compared on individual channels: CD45, CD31, PDPN, ICAM-1, and VCAM-1. The ICAM-1* column denotes values where the ICAM-1 Negative Compensation Bead control T(x) value was excluded.

**TABLE 4:** Overall chi-squared T(x) scores for controls and samples with all cells.

T(x) scores of controls					
Controls (P5 vs. P12)	T(x)	T(x)-control	Bins	Events P12	Events P5
CD45^+^ comp	9.0755	NA	240	2,453	2,458
CD31^+^ comp	41.2576	NA	140	1,485	2,455
PDPN^+^ comp	12.5797	NA	238	2,404	2,614
ICAM^+^ comp	0.0000	NA	128	1,028	2,429
VCAM^+^ comp	4.5997	NA	237	2,516	2,417
Negative comp	15.5744	NA	1,046	10,536	12,948
Unlabeled cells	230.8245	NA	707	7,636	9,187
*MAX*	230.8245				

T(x) scores of populations					
Unlabeled cells vs.	T(x)	T(x)-control	Bins	Events1	Events2
Population 1	519.9706	289.1461	640	7,060	16,823
Population 2	354.3236	123.4991	512	5,162	16,823
Population 3	300.2235	69.3990	554	5,754	16,823
Population 4	734.0909	503.2664	1,510	164,582	16,823

Population 4 vs.	T(x)	T(x)-control	Bins	Events1	Events2
Population 1	490.0456	259.2211	669	7,060	164,582
Population 2	250.9632	20.1387	512	5,162	164,582
Population 3	373.0378	142.2133	574	5,754	164,582

Population 3 vs.	T(x)	T(x)-control	Bins	Events1	Events2
Population 1	460.5908	229.7663	533	7,060	5,754
Population 2	297.2988	66.4743	512	5,162	5,754

Population 2 vs.	T(x)	T(x)-control	Bins	Events1	Events2
Population 1	320.4504	89.6259	512	7,060	5,162

Columns denote the following: T(x): The chi-squared T(x) score considering all parameters; T(x)-control: The chi-squared T(x) score minus the maximum control T(x) score; Bins: The number of bins used for analysis; Events: The number of cells in each population compared.

**TABLE 5:** Overall chi-squared T(x) scores for controls and samples with CD45^−^ cells.

T(x) scores of controls					
Controls (P5 vs. P12)	T(x)	T(x)-Control	Bins	Events P12	Events P5
CD31^+^ comp	53.8926	NA	144	1,487	2,455
PDPN ^+^ comp	17.3075	NA	233	2,412	2,618
ICAM ^+^ comp	1.6467	NA	129	1,028	2,429
VCAM ^+^ comp	7.7751	NA	229	2,499	2,417
Negative comp	22.0863	NA	775	7,864	10,458
Unlabeled cells	258.8266	NA	710	7,538	9,106
*MAX*	258.8266				

T(x) scores of populations					
Unlabeled cells vs.	T(x)	T(x)-control	Bins	Events1	Events2
Population 2	431.4109	172.5843	600	6,122	16,644
Population 3	339.2048	80.3782	514	5,182	16,644
Population 4.1	734.9185	476.0919	1,654	161,429	16,644
Population 4.2	324.9293	66.1027	330	3,370	16,644

Population 4.2 vs.	T(x)	T(x)-control	Bins	Events1	Events2
Population 2	129.9158	0.0000	290	6,122	3,370
Population 3	327.3996	68.5730	290	5,182	3,370
Population 4.1	55.6276	0.0000	290	161,429	3,370

Population 4.1 vs.	T(x)	T(x)-control	Bins	Events1	Events2
Population 2	272.5116	13.6850	609	6,122	161,429
Population 3	395.8526	137.0260	513	5,182	161,429

Population 3 vs.	T(x)	T(x)-control	Bins	Events1	Events2
Population 2	329.1090	70.2824	517	6,122	5,182

Columns denote the following: T(x): The chi-squared T(x) score considering all parameters; T(x)-control: The chi-squared T(x) score minus the maximum control T(x) score; Bins: The number of bins used for analysis; Events: The number of cells in each population compared.

### 2.8 Comparison of methods in identifying population change

Using our method of gating populations by comparing similarities among clustering algorithms across multiple dimension reduction plots, we observed a trend of population shifts between the P5 and P12 passages that mirrored the trend found with traditional gating methods (Table 6). To compare the two passages, we gated each of our identified populations by passage number (Figure 9B). CD45^+^ cells and LECs present at P5 were nearly depleted by P12. PvCs increased over time; they were nearly absent at P5 but rose to nearly 6% by P12. FRCs remained the largest population at both passages and became more homogeneous over time. MRCs decreased between P5 and P12, consistent with the trend toward homogeneity.

**TABLE 6:** Number of cells and percentages of each of our identified populations that come from each passage.

All cells						
	# of cells	% of total	# of P5 cells	% of P5 cells	# of P12 cells	% of P12 cells
Unlabled cells	16,823		9,187		7,636	
Population 1	7,060	3.87	7,052	7.81	8	0.01
Population 2	5,162	2.83	4,910	5.44	252	0.27
Population 3	5,754	3.15	366	0.41	5,388	5.84
Population 4	164,582	90.15	77,922	86.34	86,660	93.88
Total labeled cells	182,558		90,250		92,308	

CD45^−^ cells						
	# of cells	% of total	# of P5 cells	% of P5 cells	# of P12 cells	% of P12 cells
Unlabeled cells	16,644		9,106		7,538	
Population 2	6,122	3.48	5,724	6.82	398	0.43
Population 3	5,182	2.94	290	0.35	4,892	5.31
Population 4.1	161,429	91.67	75,690	90.14	85,739	93.06
Population 4.2	3,370	1.91	2,264	2.70	1,106	1.20
Total labeled cells	176,103		83,968		92,135	

**FIGURE 10:**
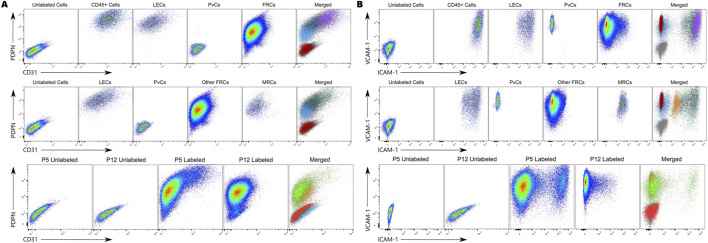
Our identified populations plotted on the traditional gating plots. **(A)** CD31 and PDPN expression, **(B)** ICAM-1 and VCAM-1 expression. Top row shows populations from the “All Cells and Compensation Beads” dataset; middle row shows populations from the “CD45^-^ Cells and Compensation Beads” dataset. Purple: CD45^+^ Cells, Teal: LECs, Red: PvCs, Blue: FRCs, Orange: MRCs. Bottom row shows the sample types: Red: P5 Unlabeled Cells, Blue: P12 Unlabeled Cells, Green: P5 Labeled Cells, Orange: P12 Labeled Cells.

Although the MRC population was not clearly distinguished from surrounding populations, it’s interesting that it would have been completely overlooked without clustering algorithms. In fact, when overlaying our identified populations onto traditional gating graphs (Figure 10), MRCs fall in the middle of the ICAM-1 axis and are only subtly visible between FRCs and LECs. The cells we would have classified as MRCs using the traditional gating method were instead classified as Lymphatic Endothelial Cells using the more robust method (Figure 10B).

**FIGURE 11:**
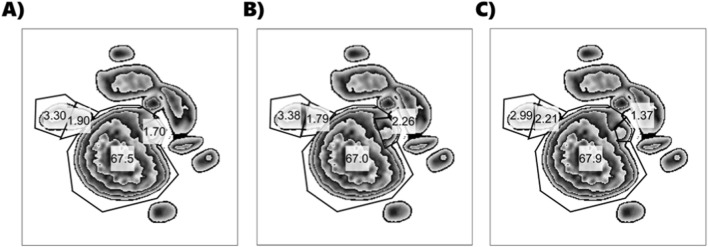
UMAP plots of the “All Cells and Compensation Beads” data set using default parameters for all algorithms. **(A)** First gates placed; **(B)** gates moved to make the largest population smaller; **(C)** gates moved to make the largest population larger. Numbers indicate the percentage of cells that fall within each gate.

Perhaps the most important comparison between these two methods is their robustness to user choices and errors. We generated ten additional arrays of plots using combinations of high, medium, and low algorithm parameters to simulate user changes (Supplementary Figure S6). We repeated our gating procedure using three criteria: 1) “normally,” following the above protocol; 2) with the goal of making the largest population smaller; and 3) with the goal of making the largest population larger (Figure 11). These three criteria allowed us to simulate a wide range of variability in gating, as if placed by separate users. Within each gating criterion, there was substantial variation in the number of cells in each population across parameter sets (Supplementary Table S1). Likewise, within each parameter set, there was substantial variation in the number of cells across the gating criteria. Overall, these variances were so large that differences between parameter sets were not significant (p > 0.05). When comparing the average variance due to gate placement in each parameter set to the variance due to gate placement in the traditional gating method, only two had higher variance; most parameter sets resulted in lower variance. Using default parameters yielded a variance of 11%, compared with the 29% variance of the traditional gating method (Supplementary Table S2). These results indicate that, under most circumstances, our method is more robust to user interpretation of gate placement than traditional gating methods. This is especially true when default algorithm parameters are used.

## 3 Discussion

Lymph Node Stromal Cells (LNSCs) are an important part of the lymph node microenvironment and play crucial roles in maintaining it. In this paper, we generated a cell line of LNSCs that preserves heterogeneity. This cell line could be useful for studying interactions among LNSCs, identifying communication signals, and working toward a self-organizing tissue-engineered lymph node. We demonstrated that care must be taken when culturing these cells over a long period of time, as the proportions among the different populations and subpopulations can change.

To assess population changes, we used flow cytometry to evaluate cell phenotypes. By measuring protein expression patterns before running a set of experiments and comparing them to those of an earlier passage, we identified shifts in the population. This is essential when working with a heterogeneous population of cells, such as most types of LNSCs, because a changing population could affect the results of experiments run over time or on different days. It is also important to recognize that in most research involving LNSCs, especially FRCs, the cells are commonly identified by two or three markers: CD31, PDPN, and perhaps CD45. This provides no information about the subpopulations present or whether those subpopulations are shifting during culture. Without understanding the population’s makeup, it can be difficult for others to reproduce the work. The power of flow cytometry over other protein expression tests, such as Western blot, lies in its ability to analyze each cell individually. This means that when a population change is detected, data is already available to investigate changes in individual subpopulations and plan future studies.

Both traditional gating and high-dimensional analysis are common methods for distinguishing populations in flow cytometry data. Using traditional gating methods, we were able to resolve large populations but struggled to draw gates where there was significant signal overlap. Without access to known positive controls that also reflected the same biological variability as our cells, accurate gating for double-positive signal was impossible. This is most clearly seen in our inability to separate LECs from FRCs using this method. The overlap between populations makes gate placement subject to bias and variability, which compounds with each subsequent gating “layer”. High-dimensional analysis addressed some of this issue. Populations could be more clearly resolved, and gates needed to be drawn only on a single level, which decreased the amount of variability due to user bias. In addition, clustering algorithms helped further inform gate placement.

However, working with machine learning algorithms for dimension reduction and clustering requires expertise to use them correctly. It can be easy for inexperienced users to blindly trust results. Proper use of these algorithms requires understanding their strengths and weaknesses, how to optimize parameters, and a good understanding of the data (Huang et al., 2022). In short, they have a steep learning curve. Additionally, working with data like ours, which has more similarities than differences between populations, would have posed a challenge for anyone. The technique we used, comparing results from multiple algorithms, looking for similar patterns among them, and validating our results, helped us make sense of our data without needing to trust the results of any single algorithm. The inclusion of controls also helped us determine when results could be trusted and how the algorithms handled biological and batch variability. Most importantly, our technique did not require expertise or optimization of the algorithms and instead relied on overall agreement for answers.

Variability is one of the biggest challenges when working with biological data, especially when the population is known to be heterogeneous. This raises questions about the distinction between mathematical and biological significance, as well as the interpretation of batch/experimental effects. The CD45 histograms for our populations are a clear example of this issue (Figure 9A). According to the Chi-Squared T(x) score, all of our populations had a significantly different CD45 signal from the unlabeled cells, even after accounting for biological variability between the controls (Table 2). Reviewing the histograms, we see that Populations 3 and 4 are clearly CD45^−^, yet they show greater CD45 variability than the control. This suggests non-specific antibody binding, which is why we ignored the statistical difference. Confirming this would require a control consisting of a similar population of cells known to have negative CD45 expression. Population 2 is a bit more confusing; it is clearly different from the other negative populations but shows a negative peak while also extending farther into the positive range. This, combined with the odd shape of its ICAM-1 histogram, suggests heterogeneity within this population. We believe some CD45^+^ cells may have been gated into this population due to the close proximity and unclear delineation between them. Even when looking only at CD45^−^ cells, we must remember that we placed a straight cutoff gate based on 99.9% of the unlabeled cells, meaning that some lower-expressing CD45^+^ cells may have fallen into this population.

Identifying rare or small subpopulations remains challenging, especially when using only a few markers. As we saw in our MRC population, a small number of cells and difficulty distinguishing it from nearby populations make it hard to detect any statistically significant difference from other populations. Although it is possible to adjust the gates until a significant population is resolved, the gate placement then becomes somewhat arbitrary. We believe this is what happened when the machine learning algorithms identified subgroups within our control populations, because these subgroups remained significantly different even after accounting for biological variability. When analyzing populations, it is essential to consider both mathematical and biological significance, which is why we gated the populations ourselves, using the algorithm results as a guide. Nevertheless, our results indicate that unresolved heterogeneity remains in our populations, pointing the way for future work.

Investigation of these populations and more precise resolution requires additional markers (Grasso et al., 2021). Using the data we gathered, we can make more informed decisions about which markers to include in a future panel. For example, can we more clearly resolve LECs using a marker unique to them? What about MRCs or other FRC subpopulations? Even without resolving additional subpopulations, we can begin investigating the cause of the shifting populations or testing whether we can change the shift. We can use functional markers to monitor changes in populations and observe their behavior using immunofluorescent microscopy. Most importantly, we now know about our population shifts and understand that our findings may reflect one subpopulation becoming more abundant than another. This knowledge alone will help improve the rigor and reproducibility of future studies of heterogeneous cell populations.

## 4 Methods

Detailed methods can be found at DOI: https://dx.doi.org/10.17504/protocols.io.81wgb9y3qlpk/v1.

### 4.1 Animal ethics statement

All mice were acquired post-mortem after euthanasia at the conclusion of other studies. No live mice were handled for the purpose of this study.

### 4.2 Lymph node retrieval and cell isolation

Inguinal lymph nodes from C57BL/6J cx32def mice were removed immediately post-mortem. The lymph nodes were separated from surrounding adipose tissue, placed into a tube of αMEM (ThermoFisher, Cat#12571071), and kept on ice. To limit cell death, cells were isolated from the lymph nodes as quickly as possible after retrieval. The following procedure was inspired by Fletcher et al., 2011. Two needles were used to gently break up the lymph node before transferring it to a tube containing 0.5 mg/mL Collagenase IV (Worthington Biochemical, Cat#LS004186) and 40 μg/mL DNAse 1 (Worthington Biochemical, Cat#LS002138) in αMEM. The tissue was digested at 37 °C while under constant agitation via gentle rotation. After 30 min, the tissue fragments were allowed to settle at the bottom and the supernatant removed. A solution of 1 mg/mL Collagenase IV and 40 μg/mL DNAse 1 in αMEM was added back to the tube before disrupting the tissue with gentle pipetting and placing it back on a rotator at 37 °C for 20 min. The tissue was further disrupted with gentle pipetting partway through the time period. After digestion was complete, any remaining fragments were broken up through gentle pipetting, and the entire contents of the tube were filtered through a 70 μm Nylon mesh. Any large pieces were gently pressed against the mesh with a syringe plunger before the mesh was washed with 3 mL media and the media was collected with the cell suspension. The cell suspension was immediately centrifuged at 470 *g* for 5 min at room temperature. The cell pellet was resuspended in 6 mL culture media containing αMEM, 10% Low IgG FBS (ThermoFisher, Cat#A3381901), and 1% Penicillin-Streptomycin then seeded into a T25 flask. The flask was incubated at 37 °C with 5% CO_2_. After 24 h, non-adherent cells were removed by very gently washing the culture surface with 37 °C PBS. Then, fresh culture media was added, and the cells were incubated until ready for immortalization.

### 4.3 Immortalization of cells

The cells were incubated until they covered around 50%–60% of the flask surface. Then, the media was removed from the flask and the cells gently washed a maximum of 6 times with warm (37 °C) PBS. The condition of the cells was checked after each wash to ensure all non-adherent cells were removed and no adherent cells began to lift from the surface. As soon as adherent cells began to retract and appear more rounded, washing was immediately stopped and 2 mL warm media with 4 mL Lenti-SV40T Lentivirus Supernatant (abm, Cat#G256) and 5.3 ug/mL Polybrene Transfection Reagent (Millipore Sigma, Cat#TR-1003-G) was added to the flask. The cells were then incubated overnight (about 12 h). In the morning, the media was removed and replaced with 6 mL warmed Lenti-SV40T Lentivirus Supernatant and 9.3 ug/mL Polybrene Transfection Reagent. The cells were incubated for 6 h before removing the supernatant and replacing it with fresh culture media. The cells were then placed back into the incubator to recover. From this point, we will refer to our immortalized cells as Lymph Node Cells (LNCs).

### 4.4 Cell culture

Each time the cells covered at least 80% of the flask surface, they were passaged to expand the population. First, the old media was aspirated, and the cells were gently washed with warm PBS. Then, just enough Trypsin/EDTA was added to cover the cells, and the flask was placed into the incubator for 3–5 min until the cells lifted. Fresh media was then immediately added to the flask to dilute the Trypsin/EDTA and wash into the cell suspension any cells stuck to the surface. The cell suspension was centrifuged for 5 min at 470xg. The cells were then counted and either seeded into flasks at a density of 1x10^4^ cells/cm^2^ or frozen in a solution of culture media with 1% DMSO.

### 4.5 Flow cytometry

#### 4.5.1 Antibody titration

Antibodies for Flow Cytometry were first titrated to determine the optimal concentration for analysis. Fluorophore conjugated antibodies were selected for CD45 (PE/Cyanine7 anti-mouse CD45 antibody, Biolegend, Cat#157205), CD31 (Brilliant Violet 605 anti-mouse CD31 antibody, Biolegend, Cat#102427), PDPN (Brilliant Violet 421 anti-mouse podoplanin antibody, Biolegend, Cat#127423), ICAM-1 (PE anti-mouse CD54 antibody, Biolegend, Cat#116107), and VCAM-1 (Alexa Fluor 647 anti-mouse CD106 antibody, Biolegend, Cat#105712). Each antibody was prepared at 2X, 1X, 1/2X, 1/10X, 1/25X, and 1/125X the recommended dilution. Three cell lines were used as positive controls: RAW 264.7 for CD45, mBMEC for CD31, ICAM-1, and VCAM-1, and our prepared cells for PDPN (determined as a suitable positive control based on preliminary data). The cells were prepared according to their typical culturing protocols. The cell pellets were washed with staining buffer two times before being resuspended to a concentration of 5-10X10^6^ cells/mL and aliquoted at 100 µL/tube. The tubes were then centrifuged, and the cells resuspended with the corresponding antibody dilution. The tubes were kept at 4 °C for 30 min then washed 3 times with staining buffer. Finally, they were re-suspended in 500 µL staining buffer and transferred to 12 × 75 mm tubes for analysis.

#### 4.5.2 Sample preparation

When preparing our cell samples, the above-described protocol was followed using 1/2X the recommended antibody concentration for each antibody. UltraComp eBeads™ Plus Compensation Beads (ThermoFisher, Cat#01–3,333–41) were prepared likewise. The following samples were analyzed: Unlabeled LNCs, Compensation Beads with anti-CD45, Compensation Beads with anti-CD31, Compensation Beads with anti-PDPN, Compensation Beads with anti-ICAM-1, Compensation Beads with anti-VCAM-1, and LNCs with all antibodies. All controls were run on both days of testing.

#### 4.5.3 Analysis

Samples were run on the BD LSR II Flow Cytometer and all Flow Cytometry analysis was performed in FlowJo™ v10.10.0 Software (BD Life Sciences). For each run, sample quality was checked using the built-in tool. Multiplets and debris were gated out of each sample and the compensation matrix was created using AutoSpill. All samples were exported with compensated parameters into a single workspace for analysis.

Statistical analysis was performed using the built-in population comparison tool, ensuring that the number of bins did not surpass 10% of the lowest number of events, then subtracting the Chi-Squared T(x) of the control population from the test value to account for biological variation.

## Data Availability

The raw data supporting the conclusions of this article will be made available by the authors, without undue reservation.
