# Supplementary material for: Resolving heterogeneity in Lymph Node Stromal Cells using high-dimensional analysis of non-optimized flow cytometry data
Source: Front Bioinform. 2026 Apr 14;6:1657030. doi: 10.3389/fbinf.2026.1657030 (PMC13121901; doi:10.3389/fbinf.2026.1657030)
Supplement: Supplementary file 1 [file DataSheet1.pdf]

Supplementary Material

1 Supplementary Figures and Tables

1.1 Supplementary Figures

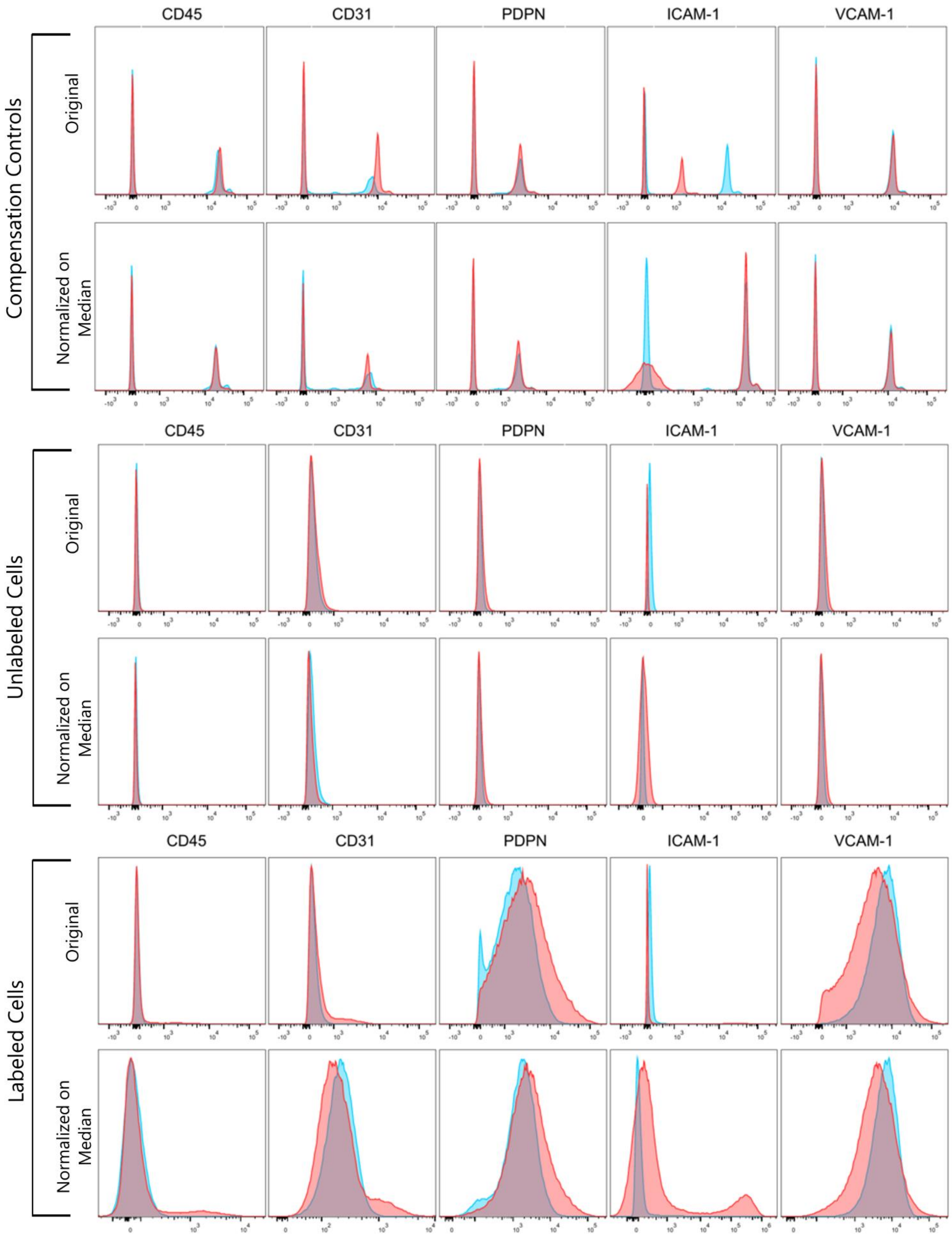

Supplementary Figure 1. Histograms showing the effect of normalization on all five channels. Red denotes P5 samples. Blue denotes P12 samples.

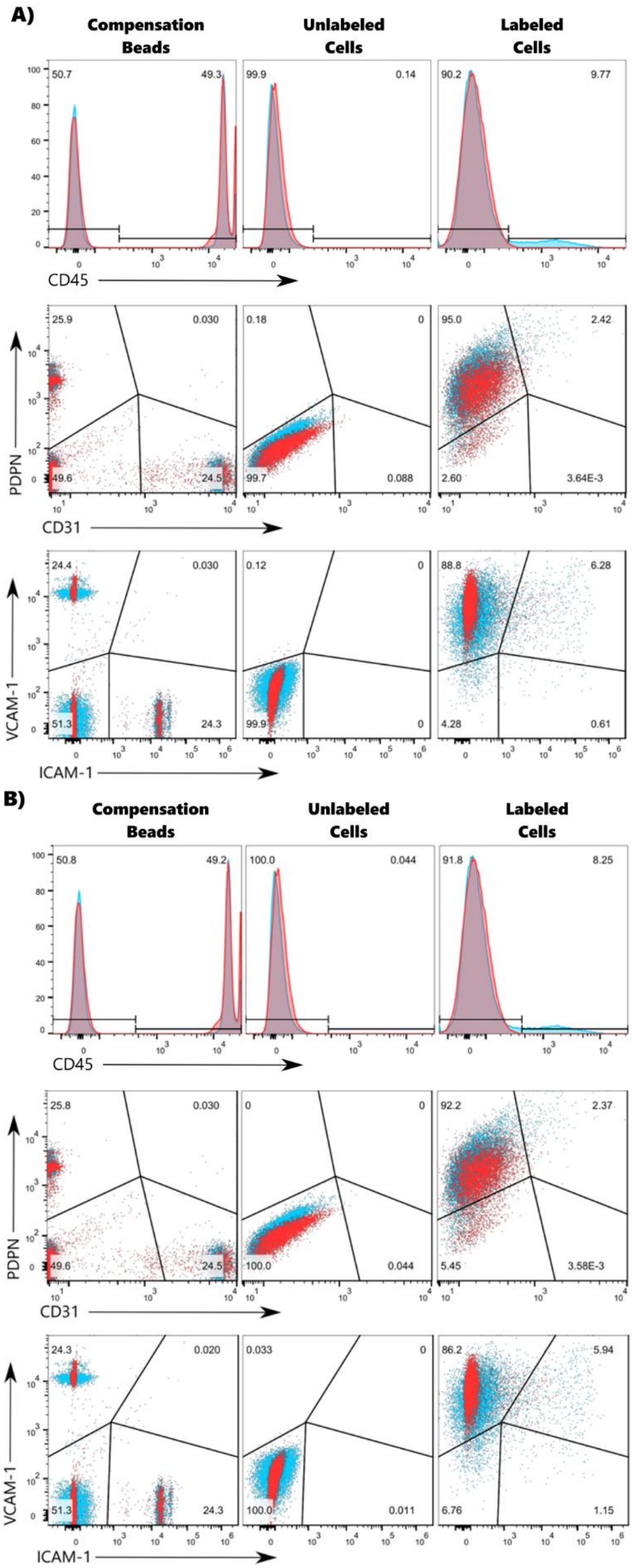
A)
Compensation Beads
Unlabeled Cells
Labeled Cells
CD45
PDPN
CD31
VCAM-1
ICAM-1
B)
Compensation Beads
Unlabeled Cells
Labeled Cells
CD45
PDPN
CD31
VCAM-1
ICAM-1

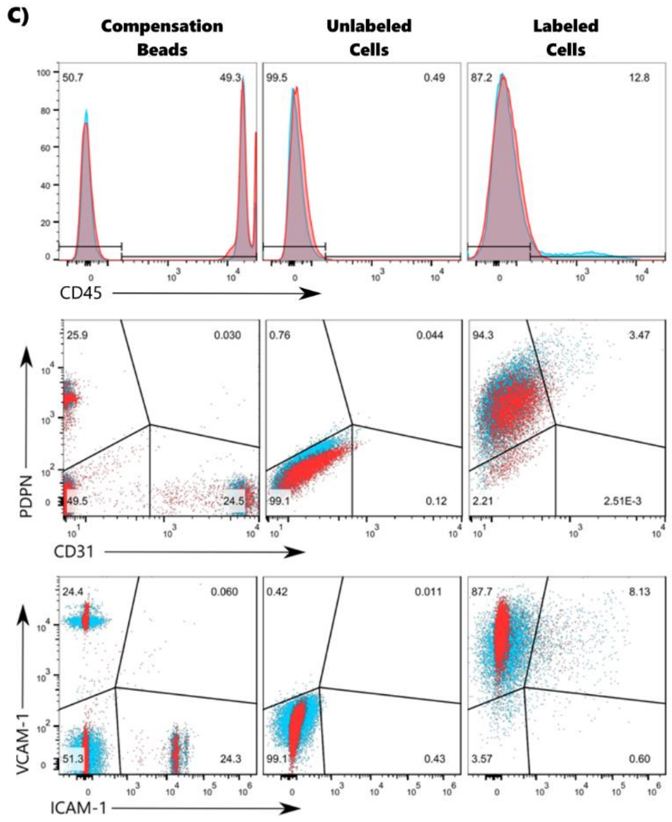

Supplementary Figure 2. Traditional gating method following three criteria: A) setting the gate as low as possible with 99.9% of the unlabeled cells within the negative gate, B) setting the gate as low as possible with 100% of the unlabeled cells within the negative gate, and 3) setting the gate so that 99.5% of the unlabeled cells fell within the negative gate. Numbers indicate the percentage of events from P5 samples falling within each gate. Blue denotes P5 samples. Red denotes P12 samples.

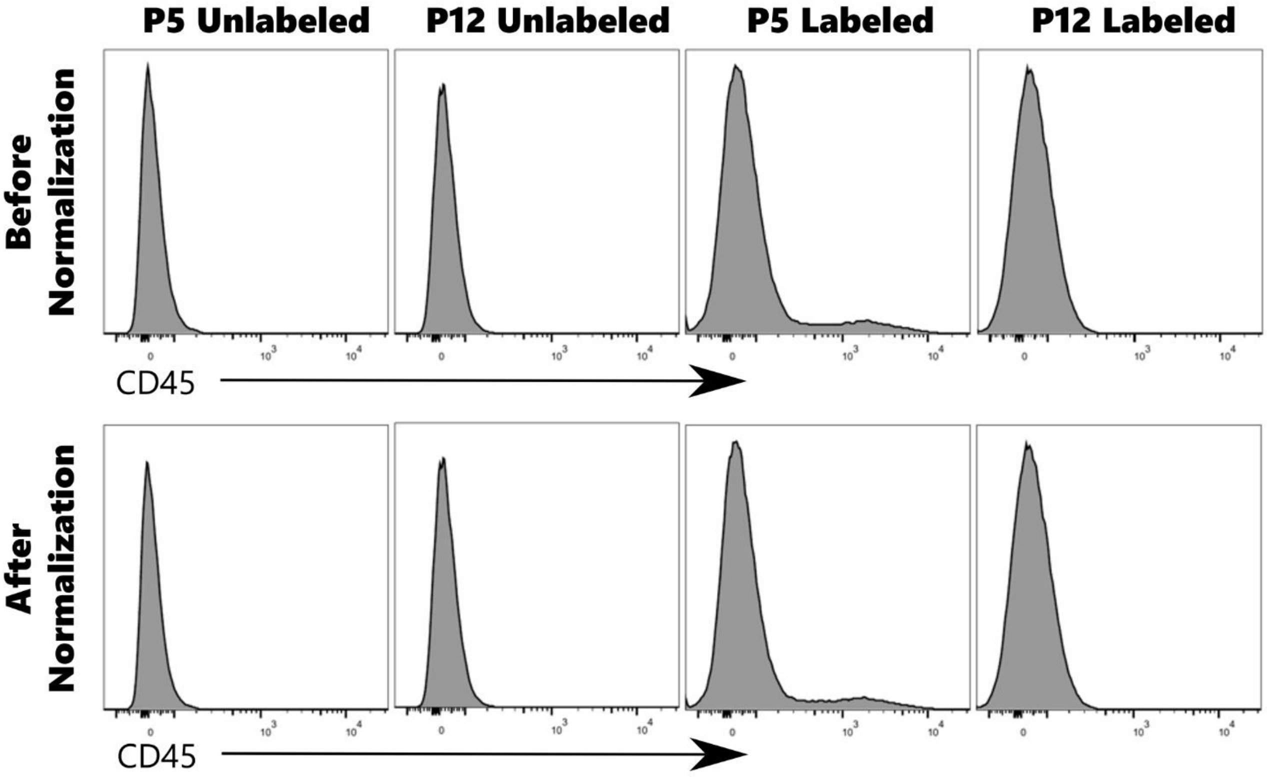

Supplementary Figure 3. Comparison of CD45 histograms used for gating before and after normalization for all four cell samples.

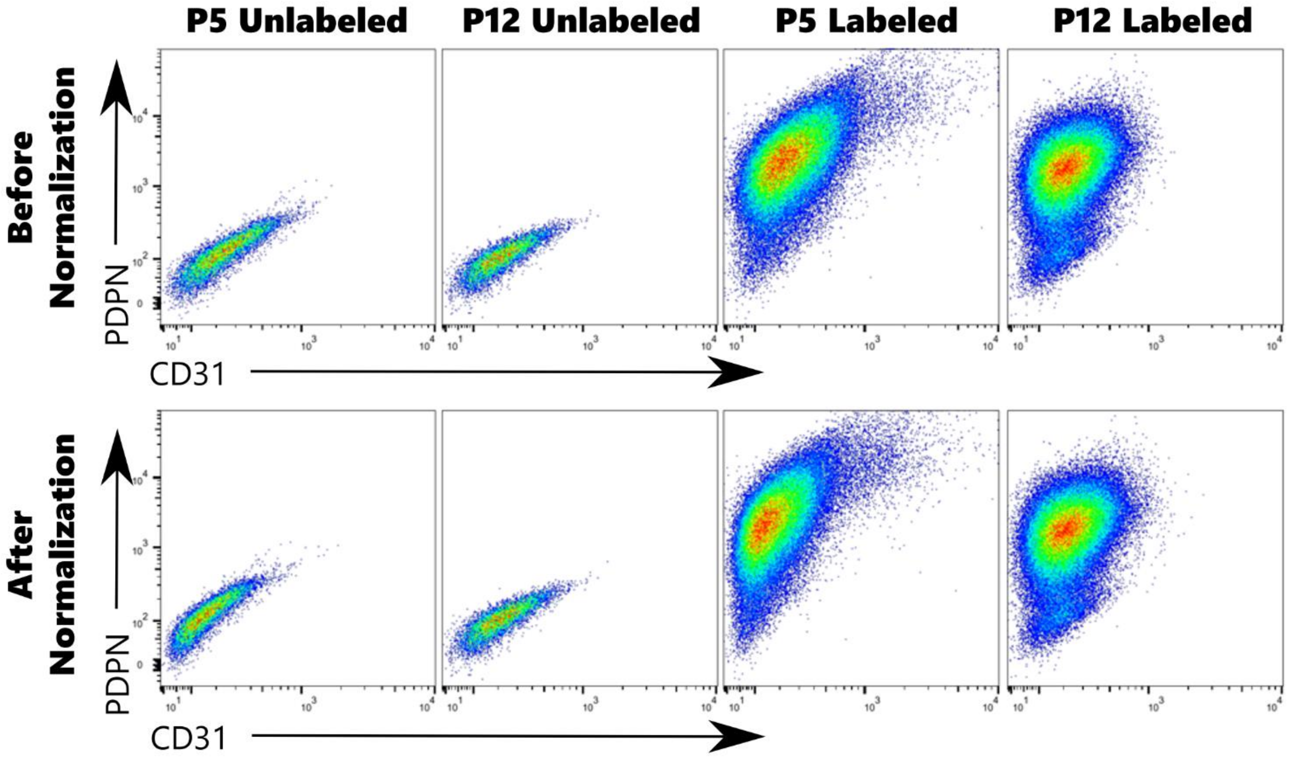

Supplementary Figure 4. Comparison of podoplanin and CD31 plots before and after normalization for all four cell samples.

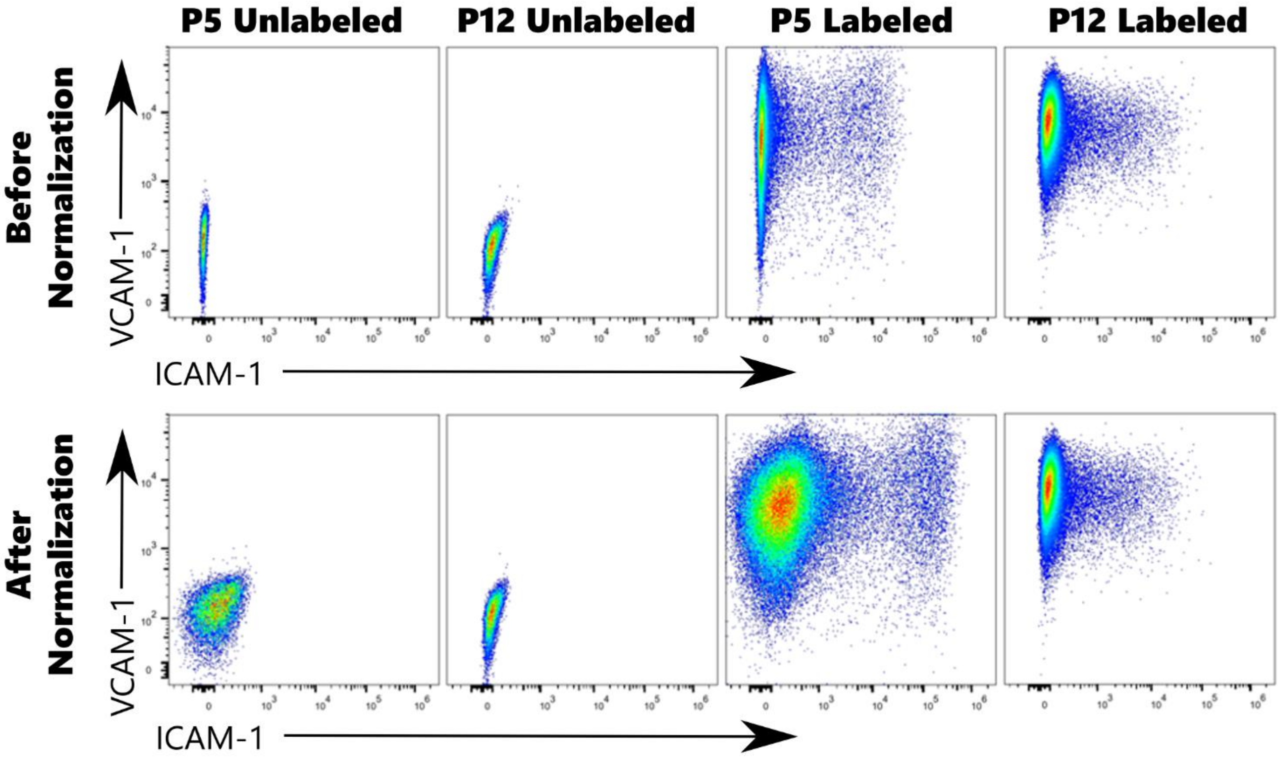

Supplementary Figure 5. Comparison of VCAM-1 and ICAM-1 plots before and after normalization for all four cell samples.

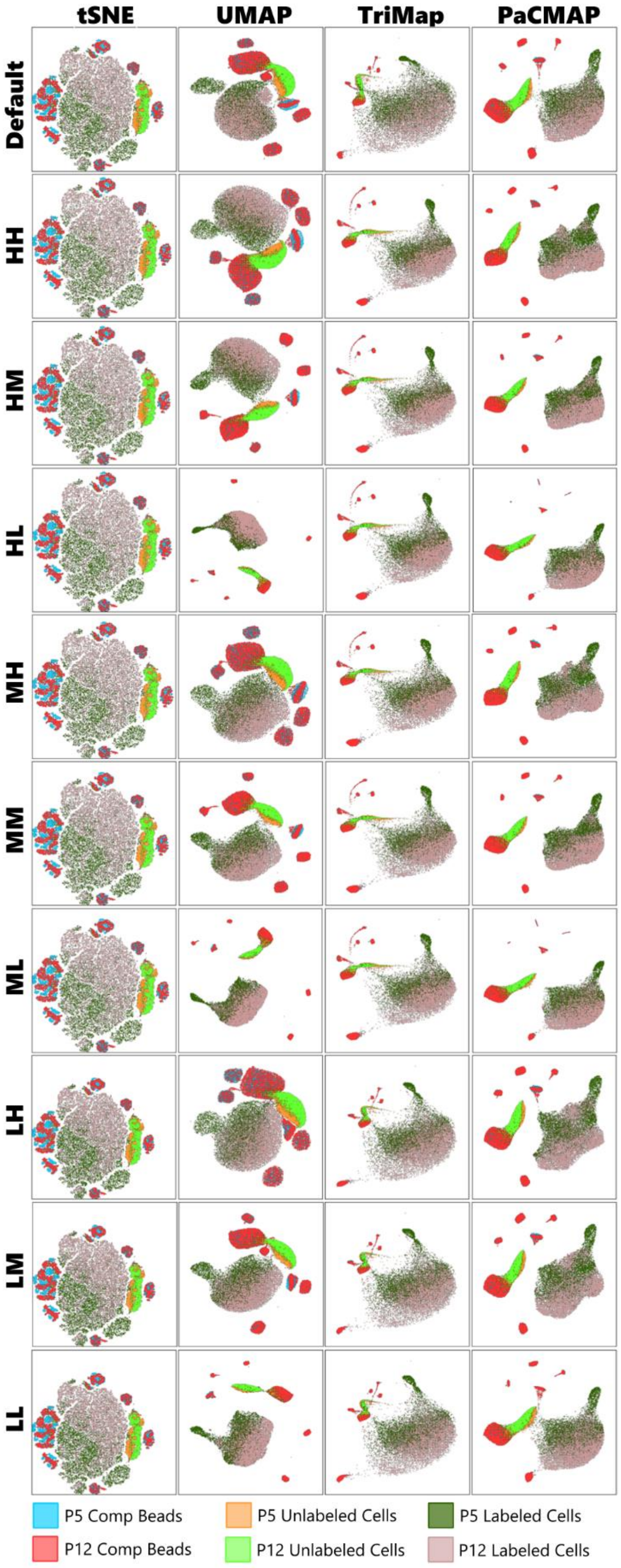

Supplementary Figure 6. Dimension Reduction plots for 10 parameter sets. DD: default settings for both K/NN/Perplexity and Min Distance/FP Ratio. High, medium, and low values for K/NN/Perplexity (First Letter) are 60, 45, and 15 respectively. High, medium, and low values for Min Distance (UMAP Second Letter) are 0.8, 0.4, and 0.02 respectively. High, medium, and low

values for FP Ratio (PaCMAP Second Letter) are 5, 3, and 1 respectively. High, medium, and low values for NN (X-Shift First Letter) are 400, 310, and 225 respectively.

1.2 Supplementary Tables

CD45+	DD	HH	HL	HM	MH	ML	MM	LH	LL	LM	Trad
Gating 1	8128	7434	8135	7899	7861	9890	9224	7728	8097	7720	9426
Gating 2	8314	6901	8135	8317	8088	9553	8573	7615	8165	8171	15463
Gating 3	7349	6901	8135	7266	6666	7978	7196	7559	7698	7013	7584
Mean	7930.333	7078.667	8135.000	7827.333	7538.333	9140.333	8331.000	7634.000	7986.667	7634.667	10824.333
SD	511.967	307.728	0.000	529.152	763.941	1020.616	1035.432	86.087	252.294	583.697	4121.427
CV	0.065	0.043	0.000	0.068	0.101	0.112	0.124	0.011	0.032	0.076	0.381
**LEC**	**DD**	**HH**	**HL**	**HM**	**MH**	**ML**	**MM**	**LH**	**LL**	**LM**	**Trad**
Gating 1	4681	5256	9298	5077	4562	6936	2639	4107	9851	3785	2736
Gating 2	4397	4492	9610	6747	6716	7896	6820	4851	10592	4981	4620
Gating 3	5436	4492	3281	4239	4406	3307	3832	3554	3526	4002	2401
Mean	4838.000	4746.667	7396.333	5354.333	5228.000	6046.333	4430.333	4170.667	7989.667	4256.000	3252.333
SD	536.998	441.096	3567.396	1276.793	1291.004	2420.405	2153.762	650.840	3883.363	637.174	1196.219
CV	0.111	0.093	0.482	0.238	0.247	0.400	0.486	0.156	0.486	0.150	0.368
**PvC**	**DD**	**HH**	**HL**	**HM**	**MH**	**ML**	**MM**	**LH**	**LL**	**LM**	**Trad**
Gating 1	4193	10373	9492	7023	4988	6359	7233	7954	6618	7170	9473
Gating 2	5566	14044	11768	9872	10045	13176	13624	10553	10323	11518	7913
Gating 3	3369	7603	5660	5702	3049	4567	4422	7025	4566	4566	16000
Mean	4376.000	10673.333	8973.333	7532.333	6027.333	8034.000	8426.333	8510.667	7169.000	7751.333	11128.667
SD	1109.873	3230.986	3086.856	2131.148	3611.947	4542.350	4715.637	1828.689	2917.784	3512.270	4290.200
CV	0.254	0.303	0.344	0.283	0.599	0.565	0.560	0.215	0.407	0.453	0.386
**FRC**	**DD**	**HH**	**HL**	**HM**	**MH**	**ML**	**MM**	**LH**	**LL**	**LM**	**Trad**
Gating 1	166138	161442	155925	163352	165681	160237	164756	163755	159044	164718	162126
Gating 2	164784	155389	153718	158228	158196	152796	154780	160401	153827	158950	155771
Gating 3	167096	164363	166043	166206	168849	167341	168114	165535	167494	167757	157781
Mean	166006.000	160398.000	158562.000	162595.333	164242.000	160124.667	162550.000	163230.333	160121.667	163808.333	158559.333
SD	1161.638	4577.185	6572.042	4042.466	5470.342	7273.151	6935.324	2606.903	6896.937	4473.414	3248.208
CV	0.007	0.029	0.041	0.025	0.033	0.045	0.043	0.016	0.043	0.027	0.020

Supplementary Table 1. Number of cells from each population using three gating criteria across ten parameter sets and traditional gating method. DD: default settings for both K/NN/Perplexity and Min Distance/FP Ratio. High, medium, and low values for K/NN/Perplexity (First Letter) are 60, 45, and 15 respectively. High, medium, and low values for Min Distance (UMAP Second Letter) are 0.8, 0.4, and 0.02 respectively. High, medium, and low values for FP Ratio (PaCMAP Second Letter) are 5, 3, and 1 respectively. High, medium, and low values for NN (X-Shift First Letter) are 400, 310, and 225 respectively.

CVs	DD	HH	HL	HM	MH	ML	MM	LH	LL	LM	Trad
CD45+	0.064558	0.043473	0	0.067603	0.101341	0.111661	0.124287	0.011277	0.031589	0.076454	0.380756
LEC	0.110996	0.092927	0.482319	0.23846	0.24694	0.40031	0.48614	0.156052	0.486048	0.149712	0.367803
PvC	0.253627	0.302716	0.344003	0.282933	0.599261	0.565391	0.559631	0.21487	0.407	0.453118	0.385509
FRC	0.006998	0.028536	0.041448	0.024862	0.033307	0.045422	0.042666	0.015971	0.043073	0.027309	0.020486
Mean	0.109045	0.116913	0.216943	0.153465	0.245212	0.280696	0.303181	0.099542	0.241928	0.176648	0.288638

Supplementary Table 2. Covariance of each population using three gating criteria across ten parameter sets and traditional gating method. DD: default settings for both K/NN/Perplexity and Min Distance/FP Ratio. High, medium, and low values for K/NN/Perplexity (First Letter) are 60, 45, and 15 respectively. High, medium, and low values for Min Distance (UMAP Second Letter) are 0.8, 0.4, and 0.02 respectively. High, medium, and low values for FP Ratio (PaCMAP Second Letter) are 5, 3,

and 1 respectively. High, medium, and low values for NN (X-Shift First Letter) are 400, 310, and 225 respectively.

# of Events	Parameter	Average Mbytes	Time Elapsed
246007	DD	1069.599821	0:49:00
246007	HH	3742.066427	4:07:00
246007	HL	4533.956731	3:56:40
246007	HM	4364.793571	3:52:10
246007	LH	1279.161266	0:44:00
246007	LL	1031.963078	0:42:20
246007	LM	1323.107933	0:43:50
246007	MH	2876.842212	2:22:18
246007	ML	2230.877266	2:19:10
246007	MM	3382.707927	2:25:50
200380	Trad	57.77777778	07:20.0

Supplementary Table 3. Computer resource usage for each analysis. DD: default settings for both K/NN/Perplexity and Min Distance/FP Ratio. High, medium, and low values for K/NN/Perplexity (First Letter) are 60, 45, and 15 respectively. High, medium, and low values for Min Distance (UMAP Second Letter) are 0.8, 0.4, and 0.02 respectively. High, medium, and low values for FP Ratio (PaCMAP Second Letter) are 5, 3, and 1 respectively. High, medium, and low values for NN (X-Shift First Letter) are 400, 310, and 225 respectively.
